# Effect of Okara Inclusion on Starch Digestibility and Phenolic-Related Health-Promoting Properties of Sorghum-Based Instant Porridges

**DOI:** 10.3390/foods14234149

**Published:** 2025-12-03

**Authors:** Adeyemi Ayotunde Adeyanju, Oluwafemi Ayodeji Adebo

**Affiliations:** 1 Centre for Innovative Food Research (CIFR), Department of Biotechnology and Food Technology, Faculty of Science, University of Johannesburg, Doornfontein 2092, Gauteng, South Africa; 2 Department of Food Science and Nutrition, College of Pure and Applied Sciences, Landmark University, Omu-Aran 251103, Nigeria

**Keywords:** sorghum, okara, fermentation, instant porridges, resistant starch, HPLC

## Abstract

This study investigated the effect of okara incorporation on modulating starch digestibility and phenolic-related bioactivity in sorghum-based instant porridges. Fermented and unfermented formulations were prepared using sorghum, okara, and their composite blends. Okara substitution at 50% reduced rapidly digestible starch by 37% and increased resistant starch by 368%. Antioxidant indicators such as 2,2-Diphenyl-1-picrylhydrazyl (DPPH) (60.22 µmol TE/g), 2,2′-Azino-bis (3-ethylbenzothiazoline-6-sulfonic acid) (ABTS) (68.33 µmol TE/g), and Ferric Reducing Antioxidant Power (FRAP) (66.09 µmol TE/g) of the 50:50 fermented composite porridge were significantly higher compared with the sorghum-only porridge. All porridges demonstrated DNA protective properties, while the HPLC profile revealed increased phenolic acids (e.g., 312.90 µg/g in fermented okara porridge) and flavonoids (e.g., 1384.21 µg/g in fermented sorghum porridge), with bioconversion of glycosylated isoflavones into aglycones. Overall, the findings highlight the synergistic benefits of incorporating okara and fermenting it in sorghum-based porridges, offering valuable insights for developing functional cereal-based products.

## 1. Introduction

The global burden of noncommunicable diseases (NCDs), chiefly cancer, diabetes, and cardiovascular conditions, continues to escalate, contributing to approximately 74% of annual deaths, or equivalent to nearly 41 million people worldwide [1]. In sub-Saharan Africa, diet-related NCDs such as cardiovascular disease, diabetes, and liver cancer are among the leading causes of mortality [2]. In recent years, this development has prompted growing interest in functional foods with enhanced nutritional and health benefits. Instant porridges, in particular, have garnered significant attention due to their convenience, affordability, and potential to deliver essential nutrients and bioactive compounds, especially among sub-Saharan African populations.

Sorghum (*Sorghum bicolor* (L.) Moench) is one of the most widely cultivated cereals in sub-Saharan Africa and is valued for its ability to thrive in drought-prone and resource-limited environments. It is high in dietary fiber, resistant starch, and polyphenols [3,4,5], which are associated with improved metabolic health. The high levels of polyphenols in sorghum, including flavonoids and tannins, have been reported to possess strong antioxidant and anti-inflammatory properties, as well as other numerous health benefits [4,6]. Despite these advantages, the utilization of sorghum in value-added food products, such as instant porridges, remains limited. Challenges include perceived lower nutritional quality and restricted consumer acceptance compared with other grains. One potential solution is to combine sorghum with nutritionally complementary ingredients or incorporate food-processing by-products with functional potential, such as okara, to improve both utilization rate and consumer appeal.

Okara, a nutrient-rich byproduct of soybean processing, is particularly rich in protein, dietary fiber, minerals, and bioactive compounds. Our earlier study demonstrated that incorporating okara into sorghum-based instant porridge formulation significantly improved protein quality and dietary fiber content [7]. Moreover, studies have shown that soluble, insoluble, and total dietary fiber extracted from okara significantly reduced serum total cholesterol, LDL-cholesterol, triglycerides, and the atherogenic index, as well as hepatic lipid accumulation in high-cholesterol mice [8]. Effects were most pronounced for the soluble fraction, primarily by suppressing hepatic lipid synthesis and enhancing cholesterol excretion. Additionally, okara contains substantial levels of isoflavones, such as genistein and daidzein, which are known for their protective effects against oxidative stress and chronic diseases, including cardiovascular disorders and certain types of cancer [9]. Thus, combining sorghum and okara in porridge formulation may yield a product with enhanced health-promoting potential by integrating the polyphenol-rich profile of sorghum with the isoflavone content of okara. For this reason, okara-enriched sorghum instant porridges present a promising approach for developing functional, nutrient-dense cereal products. However, no studies have examined how the incorporation of okara affects starch digestibility and phenolic-related health benefits in sorghum-based instant porridge formulations. Therefore, this study aimed to investigate the effects of okara addition on in vitro starch digestibility and phenolic-related wellness-supporting properties of sorghum-based instant porridges, with the aim of developing nutrient-dense, health-promoting instant food products.

## 2. Materials and Methods

Soybean and red non-tannin sorghum samples were sourced from a local market in Bodija, Ibadan, Oyo State, Nigeria, and stored at −10 °C in sealed containers to maintain dryness and coolness until use. All reagents used were of analytical grade.

### 2.1. Preparation of Sorghum Flour

Sorghum grains were cleaned manually to remove extraneous materials before milling. Whole-grain sorghum flour was produced using a milling machine (Christy Turner Ltd., Suffolk, UK) equipped with a 500-μm sieve. The resulting flour was stored at −10 °C until analysis.

### 2.2. Preparation of Okara Flour

The process illustrated by Adeyanju et al. [7] was followed in the production of okara flour. Soybeans were sorted, washed, and soaked in deionized water (1:3 *w*/*v*) for 24 h at ambient temperature, then rinsed and wet-milled to produce a slurry, from which soymilk was separated using cheesecloth. The resulting okara was oven-dried at 40 °C for 48 h, milled (Christy Turner Ltd., Suffolk, UK) to pass a 500-µm screen to obtain okara flour, and stored in airtight containers at −10 °C prior to use.

### 2.3. Preparation of Composite Flours

Composite flours were prepared by mixing sorghum and okara flours at weight ratios: 70:30 and 50:50, respectively. These specific proportions were selected based on favourable outcomes from preliminary evaluations, where blends containing less than 30% okara exhibited only modest nutritional improvement (e.g., crude protein marginally increased to 14.89–16.15% compared with 12.86% in the sorghum-only porridge), whereas formulations with more than 50% okara received low sensory acceptability scores due to intense beany flavour (overall acceptability: 4.15–4.69 on a 9-point hedonic scale) [7].

### 2.4. Instant Porridge Preparation

Fermented and unfermented instant porridges were produced following the previously described method [7]. Fermented porridges were prepared by mixing 100 g of each flour with 1 L of deionized water to form a slurry, which was incubated at 30 °C for 30–36 h until the pH reached 3.7–3.9. The fermented slurries were then cooked at 96 °C for 10 min with continuous stirring. Unfermented porridges were prepared following the same procedure, but were cooked immediately after mixing each of the flour samples with deionized water. Thereafter, the porridge samples were freeze-dried and milled using a Silver Crest Health Food Machine (Daventry, UK) to obtain a uniform particle size suitable for passage through a 500 μm sieve.

### 2.5. In Vitro Starch Digestibility

The porridge samples and raw flowers were analyzed for rapidly digestible starch (RDS), slowly digestible starch (SDS), total digestible starch (TDS), and resistant starch (RS) using the Megazyme K-DSTRS kit (Megazyme, Bray, Ireland), adapting the procedure of McCleary et al. [10] with modifications. A 1.0 g portion of each flour sample was placed in a 250 mL Fisherbrand bottle containing anti-bumping glass beads (15 pieces). Ethanol (95%, 1 mL) was added, followed by 35 mL of 50 mM sodium maleate buffer (pH 6.0) containing 2 mM CaCl_2_. The bottles were closed and maintained in a shaking water bath (Labotec, Midrand, South Africa) set to 170 rpm at 37 °C for 5 min to achieve equilibrium. Digestion was initiated by adding 5 mL of pancreatic α-amylase/amyloglucosidase solution (4 KU PAA plus 1.7 KU AMG), and incubation was continued under the same conditions. Aliquots (1.0 mL) were withdrawn at 20 min (for RDS), 120 min (for SDS; calculated as digestible starch at 120 min minus digestible starch at 20 min), and 240 min (for TDS). Each aliquot was immediately transferred into 50 mL tubes containing 50 mM acetic acid (20 mL) to stop enzymatic activity and stored at 4 °C until analysis.

Glucose determination was performed by centrifuging the aliquots at 13,000 rpm for 5 min (Eppendorf Centrifuge, Hamburg, Germany). Supernatants (0.1 mL), prepared in duplicate, were incubated with 0.1 mL amyloglucosidase (100 U/mL) at 50 °C for 30 min, followed by the addition of 3.0 mL Glucose Oxidase/Peroxidase reagent (GOPOD reagent), and incubation at 50 °C for 20 min. A mixture of 0.2 mL of 100 mM sodium acetate buffer at pH 4.5 and 3.0 mL of GOPOD reagent served as the reagent blank. Glucose standards were prepared in quadruplicate by combining 0.1 mL of a 1 mg/mL glucose solution (Megazyme, Bray, Ireland), 0.1 mL of 100 mM sodium acetate buffer (pH 4.5), and 3.0 mL of GOPOD reagent, and then incubated at 50 °C for 20 min. Absorbance was measured at 510 nm using a microplate smartReaderTM 96 (MR-9600, Accuris Instrument, Benchmark Scientific Inc., Edison, NJ, USA). Glucose standards were prepared in quadruplicate, and digestible starch fractions, including RDS, SDS, and TDS (in g/100 g sample), were calculated as described by McCleary et al. [10].

For the determination of resistant starch, 4.0 mL of the reaction mixture (after 240 min) was transferred into 15 mL centrifuge tubes containing 4.0 mL of 95% ethanol (*v*/*v*), mixed thoroughly, and centrifuged (Eppendorf Centrifuge, Hamburg, Germany) at 4000 rpm for 10 min at 0 °C. The liquid phase was discarded, and the residue was cleaned twice using 8 mL of 50% ethanol (*v*/*v*), followed by centrifugation under the same conditions. The final pellet was dissolved in 2 mL of 1.7 M NaOH on ice with continuous vortex mixing for 20 min, neutralized with 8 mL of 1.0 M sodium acetate buffer (pH 3.8), and incubated with 0.1 mL of amyloglucosidase (3300 U/mL, Megazyme, Bray, Ireland) at 50 °C for 30 min with intermittent mixing. Using a volumetric flask, distilled water was used to adjust the volume of the digested samples to 100 mL, and the mixture was thoroughly combined. A 2 mL aliquot was centrifuged (Eppendorf Centrifuge, Hamburg, Germany) at 13,000 rpm for 5 min. Duplicate portions of 0.1 mL were added to 0.1 mL of 100 mM sodium acetate buffer, pH 4.5, and 3.0 mL of GOPOD reagent, and then incubated at 50 °C for 20 min. Absorbance was measured at 510 nm using a microplate smartReaderTM 96 MR-9600 (Accuris Instrument, Benchmark Scientific Inc., Edison, NJ, USA), alongside glucose standards and reagent blanks. Resistant starch content was expressed as g/100 g sample [10].

### 2.6. Total Phenolic Content (TPC) and Antioxidant Assays

One gram of each flour sample, prepared in duplicate, was weighed into individual 50 mL beakers for the extraction of free phenolic compounds. To facilitate extraction, 10 mL of methanol with 1% (*v*/*v*) concentrated HCl was added to each sample. The beakers were then covered with foil and shaken at 200 rpm for 2 h using an orbital shaker (Stuart, Cole-Parmer Ltd., Mumbai, India). Following centrifugation (Eppendorf Centrifuge, Hamburg, Germany) (4000 rpm, 10 min at 0 °C), the supernatant was collected. The residue was re-extracted twice for 30 min each, and all the supernatants were pooled and stored at −10 °C. The same procedure was repeated using 70% aqueous acetone as the solvent.

Total phenolic content (TPC) was determined using the Folin–Ciocalteu method [11]. A UV–Vis spectrophotometer (Biochrom Libra PCB 1500, Biochrom Ltd., Cambridge, UK) was used to measure absorbance at 765 nm. The results were reported as milligrams of gallic acid equivalents (mg GAE) per gram of sample.

The antioxidant activity against ABTS and DPPH radicals was quantified using the method outlined by Adeyanju et al. [11]. ABTS + radical was generated from ABTS and potassium persulfate. Extracts (0.2 mL) were mixed with 3 mL of ABTS working solution, incubated in the dark for 30 min, and the absorbance was measured at 734 nm. For the DPPH radical scavenging assay, 0.2 mL of each extract was mixed with 3 mL of DPPH working solution, incubated in the dark at 25 °C for 30 min, and the absorbance was measured at 515 nm. Ferric reducing antioxidant power (FRAP) was determined as described by Razola-Díaz et al. [12]. For the assay, 100 μL of each extract (prepared in 1% HCl-methanol) was combined with 200 μL of deionized water and 2 mL of FRAP reagent, and then incubated at 37 °C for 30 min. The absorbance was measured at 595 nm. The results (ABTS, DPPH, and FRAP) were expressed as µmol Trolox equivalents per gram (µmol TE/g).

### 2.7. Inhibition of the Oxidative DNA Damage Assay

The protective activity of phenolic extracts against AAPH-mediated oxidative DNA damage was determined using the procedure outlined by Adeyanju et al. [6]. Extracts were diluted tenfold in 0.2 M sterile phosphate-buffered saline (PBS), pH 7.4, and subjected to gel electrophoresis (60 V, 500 mA, and 150 W) for 60 min. DNA bands were visualized using a Gel Doc EZ Imager (Bio-Rad Laboratories, Bronx, NY, USA), and their intensities were analyzed with Image Lab software (version 5.1, Bio-Rad Laboratories, Bronx, NY, USA).

### 2.8. HPLC Analysis

Duplicate portions (5 g) of each flour sample were extracted with 10 mL of methanol acidified with 1% (*v*/*v*) HCl, and the mixture was maintained under magnetic stirring for 3 h. The extracts were centrifuged at 4000 rpm for 10 min at 0 °C (Eppendorf Centrifuge, Hamburg, Germany), and the resulting supernatants were collected and stored at −20 °C in the dark until further analysis. Phenolic acids and flavonoids were quantified using reverse-phase high-performance liquid chromatography (HPLC), following the method of Kim et al. [13] with modifications. All solvents and reagents were of HPLC grade. Before HPLC analysis, extracts were filtered through 0.45 µm PTFE syringe filters and analyzed using a Waters 1525 binary pump with a Waters 2487 dual-wavelength detector (Waters Associates, Milford, MA, USA) on a YMC-Pack ODS AM-303 column (250 mm × 4.6 mm i.d., 5 µm particle size). 

The chromatographic mobile phase comprised 0.1% acetic acid in water (solvent A) and 0.1% acetic acid in acetonitrile (solvent B) under a programmed gradient: 8–10% B (0–2 min), 10–30% B (2–27 min), 30–90% B (27–50 min), 90–100% B (50–52 min), held at 100% B (52–56 min), returning to initial conditions (56–60 min). Chromatography was conducted at a controlled flow rate of 0.8 mL/min, with a column temperature maintained at 25 °C, and an injection volume of 10 µL. Detection was performed at 280 nm. Analytical-grade standards of phenolic acids—including ferulic, p-coumaric, syringic, vanillic, protocatechuic, 4-hydroxybenzoic, caffeic, sinapic, and gallic acids—and flavonoids such as rutin, fisetin, naringin, naringenin, myricetin, quercetin, catechin, hesperidin, hesperetin, and kaempferol were purchased from Sigma-Aldrich (Boksburg, South Africa). Calibration curves were constructed for each compound using standard solutions (0–200 mg/L) prepared in 0.1% acetic acid in acetonitrile. Each standard was injected singly and in mixtures (10 µL), and linear regression equations were generated by plotting the peak areas against the concentrations. Identification of phenolic compounds was based on comparison of sample retention times with those of standards, and quantification was performed using their respective calibration curves. The results are reported as µg/g sample.

### 2.9. Statistical Analysis

Results were expressed as mean ± standard deviation from replicate measurements. One-way ANOVA followed by Fisher’s LSD test was used to evaluate significant differences. All statistical analyses were performed using IBM SPSS Statistics software (v22; IBM Corp., Armonk, NY, USA). Principal component analysis (PCA) was performed using SIMCA (version 18; Umetrics AB, Umeå, Sweden) to identify patterns and visualize sample groupings.

## 3. Results and Discussion

### 3.1. In Vitro Starch Digestibility

Table 1 presents the rapidly digestible starch (RDS), slowly digestible starch (SDS), total digestible starch (TDS), and resistant starch (RS) contents of the raw flours and porridge samples formulated from sorghum, okara, and their composites. The data indicated pronounced sample-to-sample differences, indicating that starch digestibility was strongly influenced by fermentation, flour formulation, and processing conditions. The profiles of starch digestibility in the raw flours differ significantly. For instance, raw sorghum flour had RDS and SDS values of 28.06 g/100 g and 37.73 g/100 g, respectively, contributing to a TDS of 67.99 g/100 g, while the RS value was only 12.43 g/100 g. Conversely, raw okara flour exhibited substantially lower RDS and SDS contents of 2.74 g/100 g and 8.87 g/100 g, respectively, resulting in a TDS of 11.52 g/100 g and a considerably higher RS content of 38.38 g/100 g. These values highlight the potential functional value of okara in functional food formulations designed to support glycemic control. The starch digestibility parameters observed for the raw sorghum flour in this study closely align with previously reported ranges for various sorghum cultivars, specifically 16–36% for RDS, 43.04–53.81% for SDS, and 19.03–35.51% for RS [14]. These results also conform to observations reported by Taylor and Duodu [3]. Unfermented porridges exhibited higher RDS levels compared to their respective raw flours. The RDS in unfermented sorghum porridge increased to 54.76 g/100 g, representing almost a twofold rise compared to the raw sorghum flour. This increase was likely due to gelatinization and disruption of the starch matrix during cooking, as shown in the scanning electron microscopy (SEM) micrographs obtained in our earlier study [15]. Correspondingly, SDS significantly decreased (*p* < 0.05) from 37.73 g/100 g in raw sorghum flour to 21.99 g/100 g in unfermented sorghum porridge, along with a significant drop in RS content from 12.43 g/100 g to 4.71 g/100 g.

A comparable trend was observed for okara-based porridge, where the RDS content increased to 9.96 g/100 g, compared to raw okara flour, while the RS content declined significantly to 27.15 g/100 g. A similar trend was observed by Peterson et al. [5], who reported that wet cooking resulted in a substantial increase in digestible starch content, accompanied by a significant reduction in resistant starch levels. Studies have shown that the cooking process increases the ease with which starches can be digested, particularly through the process of starch gelatinization, which occurs when starch granules are heated in water. The semi-crystalline structure of native starch is disrupted, resulting in granule swelling, rupture, amylose leaching, and loss of birefringence. These structural alterations make the starch polymers more accessible to α-amylase and other digestive enzymes, thereby improving enzymatic breakdown and increasing the amount of RDS [16]. Additionally, heat treatment may disrupt or destroy bonds between starch and other food ingredients, such as proteins and lipids, which would otherwise restrict enzyme access to starch chains.

The combination of sorghum and okara in unfermented composite porridges showed significant changes in starch digestibility compared to the unfermented porridge sample from sorghum only. For example, incorporating okara at 30% and 50% into the porridge formulation resulted in a reduction in RDS of approximately 21% and 37%, respectively, while the RS content increased markedly by 232% and 368%, indicating a significant improvement in RS levels with higher okara inclusion. Additionally, increasing the okara proportion from 30% to 50% in the unfermented composite porridges resulted in a decline in RDS and TDS (from 43.31 to 34.35 g/100 g and 53.80 to 42.08 g/100 g, respectively), while RS showed an increase (from 15.66 to 22.04 g/100 g). This can be attributed primarily to the high dietary fibre content in okara, especially insoluble fibre, which creates a physical barrier that limits starch gelatinization and enzymatic accessibility. Additionally, the soy proteins and lipids in okara can interact with starch to form enzyme-resistant complexes, while its phenolic compounds can mildly inhibit the activities of α-amylase and α-glucosidase. Together, these components can reduce starch hydrolysis and contribute to a slower post-digestive glucose release.

Further changes occurred in starch digestibility due to fermentation. Fermented sorghum only porridge exhibited a slightly lower RDS value of 51.03 g/100 g as well as a lower SDS value of 18.92 g/100 g when compared to its unfermented counterpart and a slight increase in RS of 10.84 g/100 g. Also notable is the fermentation of okara porridge, where there was a greater decrease in both RDS (1.74 g/100 g) and SDS (2.73 g/100 g) compared to its non-fermented counterpart. There was also a significant (*p* < 0.05) increase in the RS value of 41.21 g/100 g. The findings suggest that fermentation-related modifications are the cause of the observed increase in RS. Overall, both okara enrichment and fermentation enhanced RS levels while reducing digestible starch fractions, suggesting improved potential for lowering glycemic response in instant porridge formulations.

The reduced starch digestibility and elevated resistant starch content observed in the fermented porridges in relation to their unfermented counterparts may be attributed to fermentation-induced structural modifications. Fermentation can promote the generation of complexes between starch and lipids, as well as starch and proteins, and induce starch retrogradation through microbial enzymatic activity and acidification. These changes result in molecular rearrangements that produce more compact and ordered starch structures, reducing accessibility to α-amylase and consequently increasing resistant starch while decreasing the rapidly digestible starch fraction [17]. This phenomenon is consistent with earlier reports demonstrating that fermentation can enhance the formation of enzyme-resistant starch fractions in cereal-based matrices [18].

Considerable differences (*p* < 0.05) in starch digestibility were also observed between the fermented composite porridges and their unfermented counterparts. Fermentation of sorghum/okara (70:30) porridge resulted in a notable reduction in RDS (approximately 17%), accompanied by increases of about 20% in SDS and 30% in RS compared to the unfermented sample. Likewise, the fermented sorghum/okara (50:50) porridge resulted in a marked decrease in RDS (approximately 30%), accompanied by substantial increases in SDS (approximately 160%) and RS (approximately 24%). These changes highlight the impact of fermentation on modulating starch digestibility, particularly in formulations with higher okara content. Furthermore, the increasing proportion of okara led to notable shifts in starch digestibility. The fermented Sorghum/okara (70:30) porridge displayed higher RDS (35.84 g/100 g) and TDS (42.56 g/100 g) values compared to the fermented sorghum/okara (50:50) porridge, which exhibited lower RDS (23.90 g/100 g) and higher RS (27.21 g/100 g). In addition, SDS was higher in fermented sorghum/okara (50:50) porridge (17.45 g/100 g), suggesting that the combined effects of fermentation and increased okara incorporation may slow starch hydrolysis, thereby potentially reducing the glycemic index.

In general, the findings indicate that both fermentation and compositing with okara have a significant impact on the digestibility of starch. Fermentation consistently reduced RDS and SDS, while increasing RS, with the effect being most pronounced in okara-based products. The addition of okara, particularly in higher amounts, increased RS content in both unfermented and fermented formulations, steadying the trend across the formulations. These findings underscore the promise of sorghum–okara composite porridges as functional foods with altered glycemic impacts.

### 3.2. Total Phenolic Content and Antioxidant Activities

The total phenolic content (TPC) along with the antioxidant activities (DPPH, ABTS, and FRAP) showed marked differences among the samples (Table 2). The TPC of raw Okara flour was significantly (*p* < 0.05) higher, with a value of 7.70 ± 0.20 mg GAE/g, compared to that of raw sorghum flour, 4.68 ± 0.16 mg GAE/g. This difference was also observed along with stronger antioxidant activities in raw okara flour (DPPH 42.06 ± 0.99; ABTS 53.47 ± 0.98; FRAP 48.34 ± 0.49 µmol TE/g). The most plausible explanation would be the rich isoflavone and polyphenolic profile of okara (Table 3 and Table 4). When the raw flours were subjected to cooking into porridge, there was a noticeable decline in TPC and radical scavenging capacity, with unfermented sorghum porridge showing the lowest TPC of 3.92 ± 0.10 mg GAE/g and the lowest value in DPPH (21.23 ± 0.47 µmolTE/g), ABTS (27.21 ± 0.45 µmolTE/g), and FRAP (25.50 ± 0.86 µmolTE/g). This suggests a potential loss of phenolic compounds during thermal processing, likely due to heat-induced structural degradation. Additionally, the reduced extractability of phenolics may result from their interaction and complexation with macromolecules within the food matrix during cooking [6]. Composite porridges exhibited intermediate values, with higher okara inclusion resulting in higher TPC and antioxidant activities.

Fermentation further improved TPC and antioxidant activities for every sample tested. The fermented okara porridge exhibited the highest overall values (TPC: 11.15 ± 0.23 mg GAE/g, DPPH: 54.39 ± 0.86, ABTS: 63.84 ± 1.49, and FRAP: 57.33 ± 1.72 µmol TE/g). This can be attributed to the liberation of bound phenolics facilitated by microbial activity during fermentation, resulting in higher extractable phenolic compounds [6].

During fermentation, lactic acid bacteria and yeasts secrete hydrolytic enzymes, particularly β-glucosidases, esterases, and phenolic acid decarboxylases, which act on the complex matrices of cereals and legumes. These enzymes cleave glycosidic and ester bonds that normally bind phenolics to cell wall polysaccharides and proteins. The hydrolysis of these conjugated forms releases free phenolics and low-molecular-weight derivatives, which are more readily extractable and exhibit higher antioxidant activity. The acidification that accompanies fermentation can further facilitate cell wall softening and matrix disruption, improving solvent accessibility to bound phenolic pools. Together, these microbial and physicochemical processes contribute to the higher total phenolic content and antioxidant capacity observed in fermented porridges compared with their unfermented counterparts.

Among the composite porridges, the fermented sorghum/okara (50:50) porridge showed the highest TPC and antioxidant activity (TPC: 9.84 ± 0.07 mg GAE/g, DPPH: 60.22 ± 0.80, ABTS: 68.33 ± 0.49, and FRAP: 66.09 ± 1.22 µmol TE/g), demonstrating the combined effect of fermentation and okara enrichment. The combined application of okara enrichment and fermentation produced a distinct synergistic improvement in both starch digestibility and antioxidant capacity, most evident in the SOFP (50:50) composite. Compared with FSP, which exhibited high RDS (51.03 g/100 g) and low RS (10.84 g/100 g), and FOP, which showed extremely low RDS (1.74 g/100 g) but very high RS (41.21 g/100 g), the 50:50 fermented porridge achieved a balanced yet enhanced profile, with substantially reduced RDS (23.90 g/100 g) and increased RS (27.21 g/100 g). This indicates that the interaction between sorghum starch and okara fibre during fermentation moderates digestibility more effectively than either ingredient fermented alone. A similar synergistic trend was observed in antioxidant properties: SOFP (50:50) exhibited higher TPC and antioxidant activities than FSP and exceeded SOFP (70:30), reflecting both okara’s phenolic contribution and fermentation-mediated release of bound phenolics. The simultaneous increase in resistant starch and antioxidant capacity highlights a synergistic enhancement that can only be achieved through the combined effects of okara inclusion and fermentation. This research demonstrates that fermentation, combined with okara enrichment, can enhance the health-supporting properties of cereal-based products.

### 3.3. Inhibition of DNA Damage

**Figure 1:**
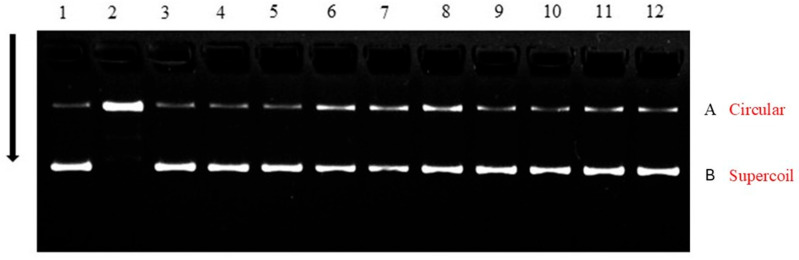
Inhibition of AAPH-induced DNA damage by raw flours, unfermented porridges, and fermented porridges from sorghum and okara. Lane 1 negative control (DNA + H_2_O); lane 2 positive control (DNA + AAPH); lane 3 (DNA + AAPH + raw sorghum flour); lane 4 (DNA + AAPH + raw okara flour); lane 5 (DNA + AAPH + 100% unfermented sorghum porridge); lane 6 (DNA + AAPH + 100% unfermented okara porridge); lane 7 (DNA + AAPH + sorghum/okara (70:30) unfermented porridge); lane 8 (DNA + AAPH + sorghum/okara (50:50) unfermented porridge); lane 9 (DNA + AAPH + 100% fermented sorghum porridge); lane 10 (DNA + AAPH + 100% fermented okara porridge). lane 11 (DNA + AAPH + sorghum/okara (70:30) fermented porridge), lane 12 (DNA + AAPH + sorghum/okara (50:50) fermented porridge).

Oxidative impairment of DNA is commonly assessed by monitoring the transformation of supercoiled plasmid DNA into open circular and linear forms, reflecting single- and double-strand breaks induced by pro-oxidants [19,20]. Figure 1 presents agarose gel electrophoretograms showing the effects of extracts from raw flours, unfermented and fermented porridges derived from sorghum, okara, and their blends on pBR322 plasmid DNA under AAPH (2,2′-Azobis (2-amidinopropane) dihydrochloride)-induced oxidative stress.

In the negative control (DNA + distilled water, Lane 1), the plasmid DNA predominantly migrated to position B in the agarose gel, indicating that it retains its supercoiled form. AAPH exposure alone (positive control, Lane 2) resulted in loss of migration, consistent with DNA accumulation at position A, indicative of the open circular form, which is less supercoiled and thus less electrophoretically mobile. This change in structure is most probably resulting from hydrogen bond dissociation by AAPH-derived alkyl peroxyl radicals [20].

Importantly, treatment with different extracts in the presence of AAPH maintained the supercoiled form of the plasmid DNA, as indicated by the migration patterns, which were similar to those of the negative control. This suggests a protective influence, most likely due to the radical-scavenging activity of the extracts’ phenolic compounds. The protective effects of phenolic-rich extracts on DNA have been previously documented [6,21].

Faint bands corresponding to open circular forms were seen in all AAPH + extract lanes, indicating that the protective effect of the extracts was partial rather than complete. This may be due to the lower protective capacity of the extracts or the presence of pre-existing open circular DNA, as minor bands were also noted in the negative control. Variability in the proportion of supercoiled form to open circular form has been observed in different batches [20].

### 3.4. Phenolic Profiling

The HPLC analysis of phenolic acids and flavonoids in raw, unfermented, and fermented sorghum and okara samples (Table 3 and Table 4) revealed significant variations in their concentrations, influenced by the type of sample, processing, and fermentation. Unprocessed okara flour (ROK) exhibited significantly higher total phenolic acid content (272.84 µg/g) relative to raw sorghum flour (245.71 µg/g; *p* < 0.05), primarily due to higher levels of p-coumaric acid (68.44 µg/g), vanillic acid (81.84 µg/g), and sinapic acid (43.73 µg/g) (Table 3). In contrast, raw sorghum flour contained higher amounts of ferulic acid (101.47 µg/g) and gallic acid (39.99 µg/g), indicating that sorghum is a richer source of these compounds. Unfermented porridges (from sorghum, okara, and their composites) generally displayed lower total phenolic acid contents compared to their raw counterparts, with unfermented sorghum porridge having the lowest (193.09 µg/g). This reduction may be due to lower extractability because of complexation with proteins and cell wall substances due to heat [6]. The reduction may also result from the covalent binding of phenolic acids to proteins at high temperatures and the thermal oxidative degradation of phenolics [22]. The blended unfermented porridges showed intermediate phenolic acid levels, reflecting the proportional contributions of sorghum and okara. The sorghum/okara (50:50) blend had slightly higher total phenolic acids (224.37 µg/g), likely due to the increased okara content, which is enriched in certain phenolic acids, such as vanillic and sinapic acids.

Fermentation significantly enhanced the phenolic acid content across all samples. Fermented porridges showed total phenolic acid concentrations ranging from 297.48 µg/g (sorghum/okara 70:30 formulation) to 312.90 µg/g (fermented okara porridge). Fermented okara porridge exhibited the highest total phenolic acid content, driven by substantial increases in p-coumaric acid (77.24 µg/g) and vanillic acid (91.33 µg/g).

Similarly, fermented sorghum porridge also showed a notable increase in ferulic acid (122.60 µg/g) and gallic acid (49.93 µg/g). The significant increase in phenolic acid content observed in the fermented porridges can be attributed to microbial-driven biochemical transformations that occur during the fermentation process. Lactic acid bacteria and yeasts produce hydrolytic enzymes, particularly β-glucosidases, esterases, and cellulolytic enzymes, that can cleave bound phenolic compounds from the cell-wall matrix, thereby increasing their release and extractability. Fermentation also disrupts the structural integrity of polysaccharides, enhancing solvent accessibility to previously entrapped phenolics. Moreover, microbial metabolic pathways can convert endogenous phenolic precursors into simpler phenolic acids through deconjugation, oxidation, or decarboxylation reactions. Together, these mechanisms account for the elevated concentrations of free phenolic acids observed in the fermented samples [6]. The fermented blended porridges exhibited phenolic profiles that reflected the combined contributions of both sorghum and okara, with the 50:50 blend showing a slightly higher total phenolic acid content (304.76 µg/g) due to the higher proportion of okara. These findings highlight the potential of fermentation to enhance the phenolic acid content of sorghum and okara-based porridges, improving their potential health-promoting properties, with okara contributing significantly to the phenolic acid pool in blended formulations.

Raw sorghum flour contains a high concentration of flavonoids, including naringenin (547.42 µg/g), luteolin (173.73 µg/g), and apigenin (143.08 µg/g), resulting in a total flavonoid content of 864.23 µg/g (Table 4). In contrast, raw okara flour is rich in isoflavones, particularly genistin (1980.18 µg/g), daidzin (349.21 µg/g), and genistein (165.28 µg/g), resulting in a significantly higher total flavonoid content of 2817.19 µg/g compared with raw sorghum flour (Table 4). Unfermented porridges generally exhibited reduced flavonoid levels compared to their individual raw flours, likely due to thermal degradation or complex formation during cooking [22]. For instance, unfermented sorghum porridge had a lower total flavonoid content (783.18 µg/g) than raw sorghum flour, with notable reductions in naringenin (from 547.42 µg/g to 408.22 µg/g) and catechin (from 54.75 µg/g to 10.26 µg/g). Similarly, unfermented okara porridge showed reductions in genistin (to 1564.05 µg/g from 1980.18 µg/g) and daidzin (to 285.47 µg/g from 349.21 µg/g) compared to ROF. The mixed sorghum-okara unfermented porridges displayed intermediate flavonoid profiles, reflecting the proportional contributions of sorghum and okara, with total flavonoids of 1368.02 µg/g and 1532.30 µg/g, respectively.

Fermentation significantly altered the flavonoid profiles, often enhancing certain compounds while reducing others. Fermented sorghum porridge exhibited increased levels of apigenin (165.42 µg/g), luteolin (317.38 µg/g), and naringenin (820.52 µg/g) compared to unfermented sorghum porridge, bringing about a total flavonoid content of 1384.21 µg/g. This suggests that microbial activity during fermentation can enhance the extraction of these compounds. Conversely, fermented okara porridge (FOP) showed a dramatic reduction in glycosylated isoflavones, genistin (55.62 µg/g) and daidzin (28.35 µg/g) but a substantial increase in their aglycone forms, genistein (467.41 µg/g) and daidzein (282.95 µg/g), indicating microbial conversion during fermentation [6]. The fermented mixed porridges reflect a balance of these trends, with sorghum/okara (70:30) formulation retaining higher levels of sorghum-derived flavonoids like luteolin (242.28 µg/g) and naringenin (582.58 µg/g), while the sorghum/okara (50:50) formulation showed elevated isoflavone aglycones such as genistein (224.35 µg/g) and daidzein (178.24 µg/g). Overall, the total flavonoid content in fermented samples (1193.50–1397.39 µg/g) suggests that fermentation may stabilize or enhance certain flavonoids while reducing others, depending on the substrate. These findings highlight the potential of fermentation to modulate flavonoid profiles, offering opportunities to optimize the potential health-promoting properties of sorghum-okara-based foods.

### 3.5. Principal Component Analysis (PCA)

**Figure 2:**
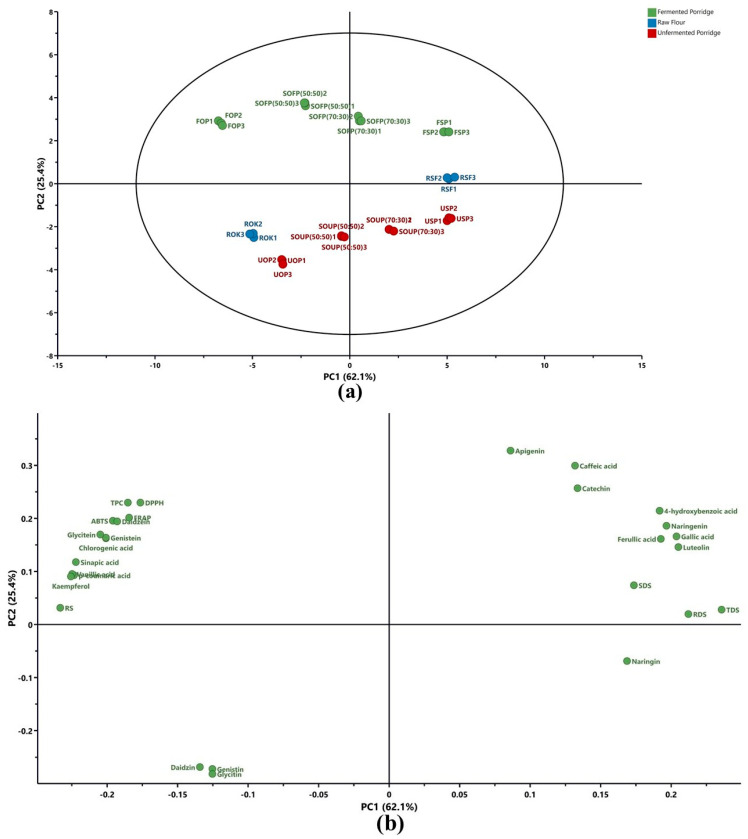
Principal Component Analysis (PCA) score (**a**) and loading plots (**b**) for sorghum-based instant porridges enriched with okara. RSF = raw sorghum flour; ROK = raw okara flour; USP = unfermented sorghum porridge; UOP = unfermented okara porridge; SOUP = sorghum/okara unfermented porridge; FSP = fermented sorghum porridge; FOP = fermented okara porridge; SOFP = sorghum/okara fermented porridge.

Principal component analysis was conducted to elucidate the multivariate relationships among treatments and measured variables, with results depicted in Figure 2a (score plot) and Figure 2b (loading plot). The first two principal components accounted for 87.5% of the total variance, with principal component 1 (PC1) accounting for 62.1% of the variance and principal component 2 (PC2) accounting for 25.4%. This high cumulative variance confirms the adequacy of the two-dimensional representation for both sample and variable interpretation within the plot. Examination of the score plot in Figure 2a manifests three observable clusters corresponding to treatment type and formulation. Fermented porridges are found within the negative quadrant of principal component 1 and the positive quadrant of principal component 2. Alternatively, unfermented porridge samples (USP, UOP, SOUP 70:30, SOUP 50:50) reside in the positive quadrant of principal component 1 and the negative quadrant of principal component 2. This distribution highlights fermentation as the principal determinant of variance along the principal component 1 axis.

The raw, unprocessed flours, ROK and RSF, are located in an intermediate region, while the component analysis further delineates additional systematic variance in the formulation and inclusion level along the principal component 2 axis. The composite blends are positioned between single-component porridges, thereby quantitatively certifying that increasing okara inclusion progressively modulated the product characteristics between sorghum- and okara-dominant profiles.

The loading plot (Figure 2b) delineates the key variables (phenolic profile, TPC, ABTS, DPPH, FRAP, RDS, SDS, TDS, and RS) responsible for the observed sample separations. Variables located on the negative side of PC1 included total phenolic content (TPC), antioxidant activities (DPPH, ABTS, FRAP), resistant starch (RS), and phenolic compounds such as chlorogenic acid, sinapic acid, vanillic acid, and isoflavones (genistein, glycitin). These attributes were strongly associated with fermented porridges, indicating that fermentation enhanced phenolic extraction and antioxidant activity while increasing RS content (Table 1 and Table 2). In contrast, unfermented porridges were aligned with variables on the positive side of PC1, including RDS, SDS, TDS, and phenolic compounds such as caffeic acid, catechin, apigenin, and naringin. These associations suggest that unfermented porridges contained higher proportions of readily digestible starch fractions and displayed a different phenolic fingerprint compared to fermented porridge samples (Table 1, Table 3 and Table 4).

The interpretation of the principal component analysis revealed two principal trajectories. First, fermentation emerged as the dominant driver, markedly elevating antioxidant capacity, as quantified by total phenolic content and various radical scavenging assays, while concomitantly enhancing resistant starch levels, which are predictors of lowered postprandial glycaemia and augmented colonic health. Second, unfermented samples exhibited elevated levels of rapidly digestible and total starch, indicating an increased glycemic potential. Incremental okara addition clearly displaced the principal component coordinates, demonstrating its capacity to modulate both starch digestibility and the phenolic profile. Collectively, the principal-component analysis substantiates the synergistic application of fermentation and incremental okara fortification as a robust platform for engineering instant sorghum porridges that exhibit attenuated starch digestibility and augmented bioactive polyphenolic fractions with putative health-promoting efficacy.

## 4. Conclusions

This study reveals that incorporating okara into sorghum, particularly at inclusion rates of 30% and 50% along with fermentation, significantly enhances the instant porridge’s potential health-promoting attributes. Fermented composite porridges exhibited notable improvements in starch digestibility, characterized by increased resistant starch and reduced rapidly digestible starch, which could potentially aid in glycemic management. Furthermore, the total phenolic content and antioxidant activities (DPPH, ABTS, FRAP) were markedly higher in all fermented porridges, especially those with okara addition. Phenolic profiling confirmed the enhanced release of phenolic acids and flavonoids, especially the conversion of glycosylated isoflavones to aglycone forms, demonstrating the bio-transformative power of fermentation. Collectively, these findings highlight the potential of integrating underutilized agro-industrial byproducts, such as okara, into cereal-based food systems to enhance their nutritional and health-promoting attributes. Further studies should focus on comprehensive sensory analysis to evaluate consumer acceptability and overall satisfaction. This is instrumental in strategizing product refinement and aiding the creation of commercially viable and public health-functional porridge interventions formulated with nutrient-dense, underutilized agro-industrial byproducts, such as okara.

## Figures and Tables

**Table 1:** Effect of okara inclusion on in vitro starch digestibility (g/100 g dry basis) of sorghum-based porridges and their flours.

Parameters	RSF (100%)	ROF (100%)	USP (100%)	UOP (100%)	SOUP (70:30)	SOUP (50:50)	FSP (100%)	FOP (100%)	SOFP (70:30)	SOFP (50:50)
RDS (g/100 g)	28.06 ^c^ ± 1.17	2.74 ^a^ ± 0.35	54.76 ^f^ ± 1.41	9.96 ^b^ ± 0.71	43.31 ^e^ ± 2.82	34.35 ^d^ ± 0.70	51.03 ^f^ ± 1.06	1.74 ^a^ ± 0.35	35.84 ^d^ ± 1.41	23.90 ^c^ ± 0.70
SDS (g/100 g)	37.73 ^f^ ± 1.18	8.87 ^c^ ± 0.98	21.99 ^e^ ± 2.46	3.73 ^ab^ ± 0.35	7.72 ^bc^ ± 1.06	6.72 ^abc^ ± 0.35	18.92 ^de^ ± 0.70	2.73 ^a^ ± 0.35	9.23 ^c^ ± 0.36	17.45 ^d^ ± 1.41
TDS (g/100 g)	67.99 ^e^ ± 1.12	11.52 ^b^ ± 0.19	75.18 ^f^ ± 0.71	14.69 ^b^ ± 0.35	53.80 ^d^ ± 1.41	42.08 ^c^ ± 1.06	70.23 ^e^ ± 0.70	4.23 ^a^ ± 0.35	42.56 ^c^ ± 1.75	38.36 ^c^ ± 1.41
RS (g/100 g)	12.43 ^bc^ ± 0.76	38.38 ^g^ ± 1.68	4.71 ^a^ ± 0.40	27.15 ^f^ ± 1.41	15.66 ^cd^ ± 0.71	22.04 ^e^ ± 1.27	10.84 ^b^ ± 0.15	41.21 ^g^ ± 1.41	20.39 ^de^ ± 1.67	27.21 ^f^ ± 1.41

Values are presented as Means ± standard deviation from replicate determinations (n = 6, two biological replicates × three technical replicates). Mean values within the same row sharing the same superscript letter are not significantly different (*p* > 0.05). RSF = raw sorghum flour; ROF = raw okara flour; USP = unfermented sorghum porridge; UOP = unfermented okara porridge; SOUP = sorghum/okra unfermented porridge; FSP = fermented sorghum porridge; FOP = fermented okara porridge; SOFP = sorghum/okara fermented porridge.

**Table 2:** Effects of okara inclusion on total phenolic content (mgGAE/g) and radical scavenging capacities (µmolTE/g) of sorghum-based porridges and their flours (dry basis).

Parameters	RSF (100%)	ROF (100%)	USP (100%)	UOP (100%)	SOUP (70:30)	SOUP (50:50)	FSP (100%)	FOP (100%)	SOFP (70:30)	SOFP (50:50)
TPC (mgGAE/g)	4.68 ^b^ ± 0.16	7.70 ^e^ ± 0.20	3.92 ^a^ ± 0.10	6.77 ^d^ ± 0.18	5.32 ^c^ ± 0.05	6.27 ^d^ ± 0.11	6.82 ^d^ ± 0.08	11.15 ^g^ ± 0.23	7.83 ^e^ ± 0.21	9.84 ^f^ ± 0.07
DPPH (µmolTE/g)	28.34 ^b^ ± 0.63	42.06 ^e^ ± 0.99	21.23 ^a^ ± 0.47	37.74 ^d^ ± 0.53	27.29 ^b^ ± 0.43	35.45 ^cd^ ± 1.73	34.06 ^c^ ± 1.12	54.39 ^g^ ± 0.86	48.28 ^f^ ± 0.55	60.22 ^h^ ± 0.80
ABTS (µmolTE/g)	32.61 ^b^ ± 1.34	53.47 ^f^ ± 0.98	27.21 ^a^ ± 0.45	47.68 ^e^ ± 1.40	33.33 ^b^ ± 0.79	43.31 ^d^ ± 0.59	39.03 ^c^ ± 0.76	63.84 ^g^ ± 1.49	55.94 ^f^ ± 1.34	68.33 ^j^ ± 0.49
FRAP (µmolTE/g)	29.12 ^ab^ ± 0.94	48.34 ^d^ ± 0.49	25.50 ^a^ ± 0.86	45.40 ^d^ ± 0.70	32.50 ^b^ ± 0.55	39.34 ^c^ ± 0.63	36.92 ^c^ ± 0.43	57.33 ^f^ ± 1.72	52.68 ^e^ ± 1.59	66.09 ^g^ ± 1.22

Values are presented as Means ± standard deviation from replicate determinations (n = 6, two biological replicates × three technical replicates). Mean values within the same row sharing the same superscript letter are not significantly different (*p* > 0.05). RSF = raw sorghum flour; ROF = raw okara flour; USP = unfermented sorghum porridge; UOP = unfermented okara porridge; SOUP = sorghum/okra unfermented porridge; FSP = fermented sorghum porridge; FOP = fermented okara porridge; SOFP = sorghum/okara fermented porridge.

**Table 3:** Effects of okara inclusion on phenolic acid contents (µg/g dry basis) of sorghum-based porridges and their flours.

Phenolic Acid	RSF (100%)	ROF (100%)	USP (100%)	UOP (100%)	SOUP (70:30)	SOUP (50:50)	FSP (100%)	FOP (100%)	SOFP (70:30)	SOFP (50:50)
p-Coumaric acids	21.99 ^b^ ± 0.96	68.44 ^g^ ± 2.31	14.32 ^a^ ± 0.80	47.51 ^ef^ ± 0.11	25.01 ^b^ ± 0.11	36.89 ^d^ ± 0.06	31.07 ^c^ ± 2.49	77.24 ^h^ ± 1.68	44.96 ^e^ ± 0.39	52.35 ^f^ ± 1.64
4-hydroxybenzoic acid	15.16 ^ef^ ± 0.79	6.02 ^ab^ ± 0.29	13.71 ^de^ ± 0.51	4.85 ^a^ ± 0.06	11.16 ^c^ ± 1.13	8.28 ^b^ ± 0.12	16.44 ^f^ ± 0.85	7.50 ^b^ ± 0.42	14.69 ^def^ ± 0.73	12.53 ^cd^ ± 0.40
Caffeic acid	14.59 ^ef^ ± 0.59	7.82 ^ab^ ± 0.64	10.91 ^cd^ ± 0.20	6.45 ^a^ ± 0.14	9.13 ^bc^ ± 0.01	7.93 ^ab^ ± 0.12	16.04 ^f^ ± 0.59	11.43 ^d^ ± 0.02	14.40 ^ef^ ± 1.22	12.84 ^de^ ± 0.01
Chlorogenic acid	2.82 ^ab^ ± 0.01	4.23 ^cd^ ± 0.50	2.47 ^a^ ± 0.00	3.65 ^bcd^ ± 0.17	2.85 ^ab^ ± 0.02	3.34 ^abc^ ± 0.18	3.37 ^abcd^ ± 0.10	5.63 ^e^ ± 0.10	3.70 ^bcd^ ± 0.25	4.40 ^d^ ± 0.25
Ferulic acid	101.47 ^f^ ± 2.64	54.39 ^ab^ ± 1.27	82.14 ^e^ ± 0.15	49.09 ^a^ ± 0.14	70.81 ^d^ ± 1.92	53.42 ^ab^ ± 0.02	122.60 ^g^ ± 3.21	58.84 ^bc^ ± 2.25	80.70 ^e^ ± 4.79	67.51 ^cd^ ± 1.26
Gallic acid	39.99 ^e^ ± 2.277	6.36 ^ab^ ± 0.48	35.59 ^de^ ± 2.34	0.23 ^a^ ± 0.08	33.91 ^de^ ± 0.19	26.76 ^c^ ± 1.82	49.93 ^f^ ± 2.39	11.19 ^b^ ± 0.10	37.58 ^de^ ± 0.18	31.13 ^cd^ ± 2.87
Sinapic acid	20.95 ^b^ ± 0.75	43.73 ^d^ ± 0.44	13.04 ^a^ ± 1.07	34.91 ^c^ ± 1.15	22.86 ^b^ ± 2.19	30.37 ^c^ ± 0.99	24.62 ^b^ ± 1.07	49.75 ^e^ ± 0.11	34.85 ^c^ ± 0.15	41.44 ^d^ ± 2.14
Vanillic acid	28.75 ^b^ ± 1.65	81.84 ^g^ ± 0.02	20.90 ^a^ ± 0.11	63.57 ^ef^ ± 0.03	44.97 ^d^ ± 0.10	57.36 ^e^ ± 0.20	35.55 ^c^ ± 0.70	91.33 ^h^ ± 2.56	66.60 ^f^ ± 1.40	82.55 ^g^ ± 4.02
Total phenolic acid	245.71 ^d^ ± 1.02	272.84 ^e^ ± 4.08	193.09 ^a^ ± 4.16	210.25 ^b^ ± 1.20	220.72 ^bc^ ± 3.03	224.37 ^c^ ± 0.83	299.61 ^fg^ ± 1.66	312.90 ^g^ ± 3.19	297.48 ^f^ ± 7.34	304.76 ^fg^ ± 0.46

Values are presented as Means ± standard deviation from replicate determinations (n = 6, two biological replicates × three technical replicates). Mean values within the same row sharing the same superscript letter are not significantly different (*p* > 0.05). RSF = raw sorghum flour; ROF = raw okara flour; USP = unfermented sorghum porridge; UOP = unfermented okara porridge; SOUP = sorghum/okra unfermented porridge; FSP = fermented sorghum porridge; FOP = fermented okara porridge; SOFP = sorghum/okara fermented porridge.

**Table 4:** Effects of okara inclusion on flavonoid contents (µg/g dry basis) of sorghum-based porridges and their flours.

Flavonoid	RSF (100%)	ROF (100%)	USP (100%)	UOP (100%)	SOUP (70:30)	SOUP (50:50)	FSP (100%)	FOP (100%)	SOFP (70:30)	SOFP (50:50)
Apigenin	143.08 ^f^ ± 2.62	109.93 ^cd^ ± 0.51	116.04 ^d^ ± 5.63	87.65 ^a^ ± 1.40	100.91 ^bc^ ± 3.53	92.28 ^ab^ ± 0.72	165.42 ^h^ ± 5.02	129.20 ^e^ ± 0.24	156.78 ^gh^ ± 0.79	144.46 ^fg^ ± 5.23
Catechin	54.75 ^d^ ± 2.30	nd	10.26 ^b^ ± 0.47	nd	7.52 ^b^ ± 0.42	4.53 ^a^ ± 0.35	78.01 ^e^ ± 2.49	nd	60.43 ^d^ ± 2.49	46.38 ^c ±^ 2.49
Daidzin	nd	349.21 ^h^ ± 2.41	nd	285.47 ^g^ ± 3.39	95.44 ^e^ ± 0.57	172.76 ^f^ ± 0.82	nd	28.35 ^c^ ± 4.16	9.39 ^a^ ± 0.21	16.96 ^b^ ± 0.52
Daidzein	nd	92.04 ^d^ ± 2.41	nd	65.04 ^c^ ± 3.39	21.96 ^b^ ± 0.57	34.99 ^b^ ± 1.78	nd	282.95 ^f^ ± 7.79	102.93 ^d^ ± 2.60	178.24 ^e^ ± 5.20
Genistin	nd	1980.18 ^f^ ± 11.18	nd	1564.05 ^e^ ± 5.39	575.01 ^c^ ± 4.40	774.20 ^d^ ± 22.01	nd	55.62 ^b^ ± 5.51	15.84 ^a^ ± 0.22	29.69 ^ab^ ± 1.32
Genistein	nd	165.28 ^e^ ± 3.91	nd	109.35 ^c^ ± 7.06	33.54 ^a^ ± 4.98	58.99 ^b^ ± 8.56	nd	467.41 ^g^ ± 8.59	142.75 ^d^ ± 0.83	224.35 ^f^ ± 7.34
Glycitin	nd	26.07 ^f^ ± 0.76	nd	22.39 ^e^ ± 0.46	7.34 ^c^ ± 0.02	15.71 ^d^ ± 0.14	nd	1.31 ^b^ ± 0.02	0.31 ^a^ ± 0.02	0.55 ^a^ ± 0.00
Glycitein	nd	88.25 ^d^ ± 0.76	nd	65.91 ^c^ ± 0.46	22.57 ^a^ ± 1.34	40.31 ^b^ ± 0.88	nd	218.33 ^f^ ± 8.80	78.39 ^cd^ ± 4.40	143.35 ^e^ ± 8.31
Kaempferol	nd	6.24 ^e^ ± 0.25	nd	4.20 ^c^ ± 0.19	1.90 ^a^ ± 0.13	2.65 ^b^ ± 0.12	nd	10.34 ^f^ ± 0.14	4.00 ^c^ ± 0.07	5.29 ^d^ ± 0.09
Luteolin	173.73 ^bc^ ± 2.63	nd	231.35 ^d^ ± 5.79	nd	188.65 ^c^ ± 3.35	105.76 ^a^ ± 0.39	317.38 ^e^ ± 11.59	nd	242.28 ^d^ ± 3.21	157.92 ^b^ ± 5.81
Naringin	33.44 ^f^ ± 1.08	nd	17.32 ^e^ ± 0.11	nd	12.61 ^d^ ± 0.08	8.23 ^c^ ± 0.28	2.88 ^b^ ± 0.05	nd	1.70 ^ab^ ± 0.11	0.95 ^a^ ± 0.03
Naringenin	547.42 ^d^ ± 10.37	nd	408.22 ^c^ ± 24.92	nd	300.56 ^b^ ± 6.01	221.88 ^a^ ± 0.10	820.52 ^f^ ± 18.52	nd	582.58 ^d^ ± 16.72	389.38 ^c^ ± 11.64
Total Flavonoids	952.42 ^b^ ± 19.01	2817.19 ^f^ ± 18.14	783.18 ^a^ ± 24.38	2204.07 ^f^ ± 21.75	1368.02 ^d^ ± 20.39	1532.30 ^e^ ± 14.36	1384.21 ^d^ ± 27.62	1193.50 ^c^ ± 0.44	1397.39 ^d^ ± 22.82	1337.51 ^d^ ± 16.62

Values are presented as Means ± standard deviation from replicate determinations (n = 6, two biological replicates × three technical replicates). Mean values within the same row sharing the same superscript letter are not significantly different (*p* > 0.05). nd = not detected; RSF = raw sorghum flour; ROF = raw okara flour; USP = unfermented sorghum porridge; UOP = unfermented okara porridge; SOUP = sorghum/okra unfermented porridge; FSP = fermented sorghum porridge; FOP = fermented okara porridge; SOFP = sorghum/okara fermented porridge.

## Data Availability

The data that support the findings of this study are available from the corresponding authors upon reasonable request.
